# Reactions to Ingroup and Outgroup Deviants: An Experimental Group Paradigm for Black Sheep Effect

**DOI:** 10.1371/journal.pone.0125605

**Published:** 2015-05-06

**Authors:** Marika Rullo, Fabio Presaghi, Stefano Livi

**Affiliations:** Dipartimento di Psicologia dei Processi di Sviluppo e Socializzazione, Sapienza University of Rome, Rome, Italy; Brock University, CANADA

## Abstract

In the classic black sheep effect (BSE) an ingroup deviant member is usually evaluated more negatively than the corresponding outgroup deviant. This effect is usually obtained by using scenarios and asking people to imagine the situation as vividly as possible. The present study proposes a new method to investigate the BSE by considering the behavioral and physiological reactions to unfair behavior (aggressive game behavior) in a realistic experimental group-setting. The study involved 52 university students in a minimal group setting who performed a modified version of the competitive reaction time (CRT) task adapted to be played in groups of four people. The classic BSE was replicated for evaluation but not for the behavioral reactions (retaliate to aggression) to deviants. More interestingly, a negative relationship emerged in the ingroup deviant condition between the level of behavioral derogation and the systolic blood pressure level.

## Introduction

Based on literature on the *black sheep effect* (BSE), people tend to derogate a negative ingroup member more than a similar outgroup member in order to protect the group from the threat that the deviant poses to their social identity [1] [2] [3] [4] [5] [6] [7]. Social identity theory (SIT) [8] suggests that people derive the central core of their identity from groups they belong to and that the higher the identification with the group, the higher is the tendency of people to express ingroup favoritism for enhancing their self-esteem [9] [10] [11]. In this sense the BSE is also considered a sophisticated form of ingroup favoritism as derogation represents a way to maintain the group at a certain distance from the negative image that may derive from the deviance of a member [1]. Similarly, other researchers [12] [13] [14] also found the identification to have a great impact on the judgments of deviant ingroup members. However, not all the actions of a deviant ingroup member have equal implications for the other members’ social identity. As shown by previous research [15] [16] [17] [18], people are generally unaffected by threats in domains not related to social identity. Hence, only when the social identity is at stake does identity threat trigger the BSE.

In the same vein, the subjective group dynamics approach (SGD) [19] [20] [21] [22] suggests that people tend to derogate those members showing immoral positions and behaviors that deviate from the prescriptive group norms. The derogation is reflected in the degree of rejection elicited by dissenters representing a threat to the group’s social reality [23] [24]. The subjective reality of the group’s norms and the adhesion to these standards provide certainty about the “right way” to behave, and hence are necessary for the members to maintain a positive social identity [25].

However, there has not been enough investigation of actual behaviors related to BSE, instead past research has relied on written scenarios about a deviant member and self- report measures [26] [12]. In particular, behavioral reactions have been investigated only in terms of efforts to persuade the deviants to become normative [27] [28]. Furthermore, the measure of derogation itself has also been investigated exclusively using self-report scales [19]; for a review, see [29]. Therefore, given the important implications of the BSE, the need to investigate this phenomenon is evident using an experimental paradigm that allows both self-reported and behavioral measures toward the deviant member in a more realistic and ecologic setting. For example, in a study Schachter [30] showed how deviants were more likely to be assigned to unattractive jobs, demonstrating that the reactions to deviants involve not only cognition (i.e., evaluations or judgments) but also a behavioral response.

In the present study our aim is [1] to propose a new manipulation of the deviant member using an experimental group-setting procedure and [2] to measure the reaction to deviant actions in terms of both behavioral and self-reported responses. Four participants, in a minimal group setting, were asked to play a competitive reaction game against the other members of the group. Losing the competition involves hearing a noisy sound whose level is decided by the winner. This group task was adapted from one of the most widely used laboratory tasks for measuring a person’s physical aggression toward the person they are playing with, i.e., the competitive reaction time task (CRT task) [31]. In the group-modified version of CRT task (the G-CRT task that is available upon request^)^, losers have the possibility of returning the aggression they receive from winners even if this involves breaking a shared group norm.

We suggest that this paradigm is important in order to understand whether the BSE may be extended to forms of ingroup derogation based on behavioral response to deviant members, beside the classic judgmental bias. We hypothesize that a behavioral derogation of negative members as well as the aggressive reaction in response to their deviance is not used by those members who want to preserve a positive social identity. As a matter of fact, derogation of deviants could be viewed as normative [32] [4]. Therefore, group members choose derogation of deviants also in order to preserve their normative position (self-presentation bias; see review [33]). At the same time, not all the responses to deviants are successful in meeting the members’ needs and in many cases it is also impossible to accomplish these needs as in the study by [21], where members failed to expel the deviant from the group. The underlying idea is that the BSE is mostly observed through evaluation because this kind of derogation does not involve an explicit breakup of the group norm (like behaving unfairly against a member when another ingroup member is present) and at the same time is a good way to achieve a positive social identity. This response represents a symbolic exclusion of the deviant and is useful for restoring group consensus [34].

That is, derogation does not indicate if the offended member would refrain from breaking the norm or rather is more prone to respect the norm. On the contrary, when retaliation implies breaking the group norm the offended member has to face a double-edged sword dilemma, since punishing a defector using the same “arm” (i.e., by endorsing the same behavior for which the deviant has to be punished) implies becoming, in turn, a deviant to the group norms. These unfair behaviors fall under the examples of prescriptive norms designed by the subjective group dynamics model and considering, among others, “honesty” and “solidarity” as kinds of moral conventions [35].

Furthermore, given that the unfair behavior may be perceived as an aggressive behavior, we also intend to monitor the implicit and non—controlled reaction of the offended member to an aggressive partner during the experimental task by measuring the blood pressure (BP) levels, which has been already used as a general marker of physiological arousal [36] [37] [38]. In particular, it was found that when students are deliberately angered, higher levels of systolic blood pressure was observed in response to the provocation and that the residual systolic blood pressure showed a significant reduction when students have the opportunity to reply (verbally or physically).

Our aim is not only to investigate whether the internal state of group members (via BP arousal) varies when facing an ingroup or an outgroup deviant, but also in studying whether the BP reaction of people varies when they are asked to not retaliate to unfair behavior endorsed by ingroup (as opposed to outgroup) members. Hence, we suggest that respecting the norm could elicit a higher activation when people face an unfair ingroup deviant in virtue of the struggle of the decision to not punish (even if it was the first choice) in contrast to the less demanding decision regarding the punishment of the outgroup deviant. Finally, as one of our main aims in the present study is to examine the effects of ingroup/outgroup manipulation in an experimental group setting, and given that the degree of identification with the group is considered one of the most important moderators of the BSE [1] [14] [12], the present study involved real group interactions in which participants could be realistically identified.

### Main Hypotheses

Based on these premises we formulate the following hypotheses:

H1) As a result of the effect of target manipulation, we expect that participants derogate their opponents when they deviate from the norm (deviant) more than opponents that do not deviate from the norm (normative). Moreover, as predicted by the BSE, we hypothesize ingroup deviant opponents to be more negatively depicted than their outgroup deviant counterparts.

H2a) However, we expect participants to not retaliate to the opponent's norm-breaking aggressive behaviors in order to obey the shared norm that suggests being not aggressive. Therefore, we expect to not find any significant differences in the average aggressive behavior (sound levels) between normative and deviant opponents.

H2b) We also expect that the norm will also hold (i.e., no differences in the average aggressive behavior) when the participants have to deal with ingroup as opposed to outgroup adversaries. So we expect to find no significant main effect of membership (ingroup vs. outgroup) on the Noisy Sound level.

H3) Furthermore, we also expect to find specific effects in the implicit reaction as a function of the target manipulation (normative vs deviant). In particular, we expect a significantly higher physiological activation (in terms of BP) in the deviant partner game, but not in the normative games, in response to the provocative aggressive behavior.

H4) Finally, we suggest that, since any tendency to retaliate against unfair (rule-breaking) behavior should be inhibited (see H2a), we expect that the more participants inhibit aggressive reaction the higher their SBP levels should be, as predicted by other previous studies [36] [37] [38].

## Materials and Methods

### Participants

Assuming a medium effect size f = 0.25, a Type-1 error of α = 0.05 and power of 1-ß = 0.90, for the basic CRT task with three repeated games (with an average correlation of about 0.3) and a factor with two conditions (ingroup vs. outgroup) we match a minimum optimal sample size of about 50 participants [39]. A total of 49 students (70% women) attending a course in Psychology (36%) or in Educational Science (64%) with a mean age of 22.8 years (SD = 2.92) were involved in the study.

### Procedure

**Fig 1:**
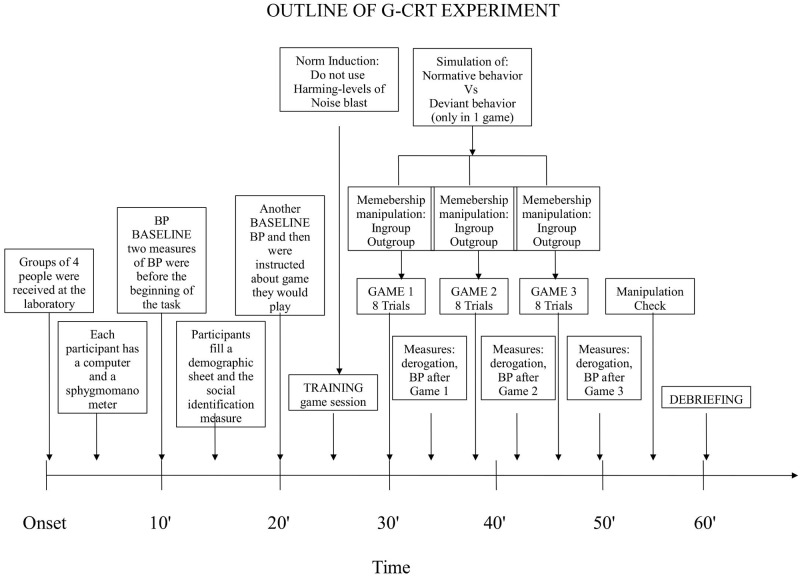
Experiment outline.

The procedure is outlined in Fig 1. Participants were received at the laboratory in groups of four people; they were seated at the four corners of the room, in such a way that nobody could see any other player. Each participant had a computer and a sphygmomanometer, which the experimenter attached to the wrist of the participant’s non-dominant hand. Participants were instructed in the correct use of the device and were invited to take two measurements of their BP before the beginning of the task (the mean score represents the baseline). Participants were then informed that during the task they would be prompted by the computer to take a measurement of their BP.

Participants first filled a demographic sheet and then answered the items on social identification by indicating their university major (i.e., Psychology or Educational Science). At the end of this first part, participants were asked to assess their diastolic and SBP and were then instructed about the game they were going to play (i.e., a modified version of the competitive reaction time task; [40]). A training game session preceded the real task to ensure that all participants understood the instructions. Before each game session, the researcher warned participants not to use the maximum levels of punishment (from 8 to 10 corresponding to a noise blast ranging from 95 to 110 dB) in order to avoid extreme suffering on the part of the opponent. This information was the priming of the norm that all participants were recommended to observe during the game. To make the norm more salient within the group, the experimenter invited participants to try the noisy sound at the maximum volume. Participants were told that they would play in turn against each one of the other students present in the laboratory using the intranet connection between computers. In fact, the computer simulated all opponents of the three games. Only one of these simulated opponents endorsed particularly punishing behaviors (deviant, i.e. from 8 to 10 noise blast level) while the others simulated non-punishing (normative, i.e. below 8 noise blast level) behaviors. Depicting the opponent with a “normative” or “deviant” punishing behavior represented the manipulation of target. The order of the deviant in the three games was systematically varied. As none of the participants knew which major the other members of the group attended, the opponent’s membership was simply determined by matching the same university major as that of participant (ingroup) or a different one (outgroup). This priming represented the membership manipulation of the deviant. Thus the simulated opponents’ group composition was the same for all participants and involved one “deviant” (ingroup–outgroup) opponent and two “normative” (ingroup) opponents. The presence of the two normative opponents and just one deviant along with experience of “playing in a group” had the sole function to make the competition as realistic as possible. Participants were randomly assigned to one of the groups and then to one of the two experimental conditions, in order to avoid groups of students who were known to each other.

**Fig 2:**
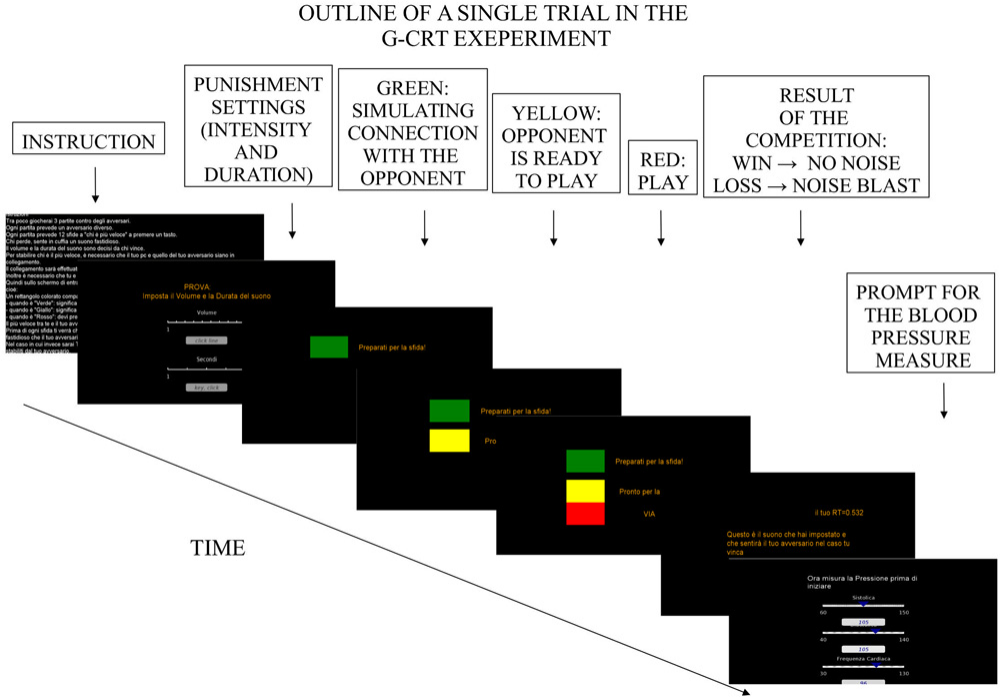
Trial outline.

To reinforce the students’ belief that the game was a real competitive situation, they were also told to wait for a green light appearing on the screen before each game session, indicating that the computer was now connected to one of the other PCs present in the laboratory (Fig 2). Participants were also told that the game software would randomly select one of the players and that no information about the game partner would be given. After each game participants were asked to rate their opponent’s behavior as well as to take his/her own diastolic and SBP (game reaction pressure). Finally at the end of the three games, participants were debriefed and dismissed and a last assessment of pressure was taken (recovery pressure). The present study was approved by the local Ethical Committee of the Department of Psychology of Social and Developmental Processes, Sapienza University of Rome. All participants signed the informed consent before being involved in the experiment.

### Measures

#### Group identification

Identification was measured using the identity scale [41]. An example of an item is “When I talk about my major, I say ‘we’ than ‘they’”. Each of the six items was measured on a 7-point scale, ranging from 1 “Strongly Disagree” to 7 “Strongly Agree” (α = .79).

#### Evaluation of target opponent

Evaluation was by five statements with a semantic differential (*pacific*-*aggressive*, *supportive*-*hostile*, *predictable*-*unexpected*, *justifiable*-*unjustifiable* and *tolerable*-*intolerable)*. The mean score obtained for each partner game was then used as a measure of devaluation (reliability of the measures of the three opponents behaviors was respectively: α(game 1) = .80; α(game 2) = .82; α(game 3) = .88).

#### Competitive reaction time (CRT) task

The competitive reaction time task is based on the aggression paradigm developed by [40] Taylor (1967), which requires participants to compete against a simulated opponent on a reaction time task. Participants are asked to compete against another person in a series of eight reaction time trials for a total of three games. The loser of each trial receives a punishment, a noisy sound of pre-specified duration, immediately after losing the trial. Before each trial participants set the sound (ranging from 0, corresponding to 60 dB, to 10, corresponding to 110 dB) and duration levels (ten possible choices, ranging from 0 seconds to 5 seconds) of the punishment. As the opponent is simulated, all the reaction tasks in terms of wins and losses are predetermined as well as the intensity and duration of the punishment chosen by opponent. The level of noisy sound chosen by the subject for each game is considered as the proxy of aggressive behavior.

#### Physiological measures

SBP and diastolic blood pressure (DBP, in mmHg) were obtained with a clinically tested wrist-cuff device (OMRON RX Genius 637IT R7) with a built-in position sensor that helped participants in finding the optimal arm position for the measure of BP. Mean Arterial Pressure (MAP) was calculated as MAP = [0.33*(SBP-DBP)+DBP] [42]. All SBP, DBP and MAP will be used as manipulation check, while SBP will be used as markers of the reaction to the unfair behavior.

## Results

### Deviant vs. Normative Reactions

**Fig 3:**
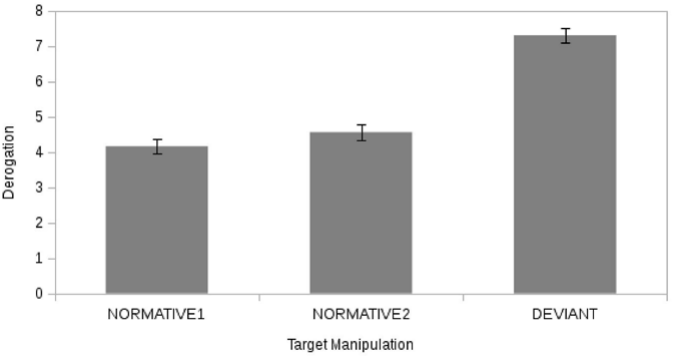
The effect of target manipulation on derogation. Derogation as function of target opponent (“deviant” vs. “normative 1” vs. “normative 2”).

As a control, before the task, participants of both groups (ingroup = 26; outgroup = 23) did not differ with respect to the level of identification (t (47) = 446, p = .66). As a manipulation check of deviant manipulation, we verified the “Evaluation of target opponent” by performing two ANOVAs: one to test the effect of repeated target manipulation (normative 1 vs. normative 2 vs. deviant) and the other to test the emergence of the classic BSE on deviant evaluation functioning of membership (ingroup vs. outgroup). As the membership manipulation is located only in the deviant partner condition, we only inspected the interaction effect of deviance and membership without investigating the membership effect interacting with the normative target. Finally, as the effect of derogation may depend on the level of identification, this factor was treated as covariate in both ANOVAs. The same design analysis was then replicated for the level of noisy “Sound” within the CRT task. The effect of target manipulation (Fig 3) was significant (F (2, 46) = 4.46, p = 0.02, η^2^_PARTIAL_ = 0.17) with the deviant opponent rated as significantly (Sidak post-hoc test) more negative (M = 7.3, p < 0.01) than the two normative opponents (respectively: M_1_ = 4.2; and M_2_ = 4.5) (H1) while the two normative opponents did not differ (p = 0.36). The identification had no significant effect on the evaluation of the opponent (Identity: F(2, 46) = 0.09, p = 0.92, η^2^_PARTIAL_ = 0.004).

**Fig 4:**
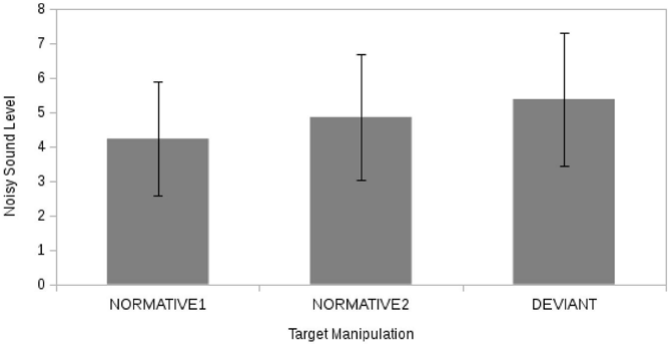
The effect of Target manipulation on reaction. Noisy Sound Level as function of target opponent (“deviant” vs. “normative 1” vs. “normative 2”).

However there was no effect of target manipulation on aggressive behavior as measured by the level of noisy Sound (H2a) (Fig 4) (F (2, 46) = 0.30, p = 0.74, η^2^_PARTIAL_ = 0.01) with the two normative opponents (M1 = 4.23; M2 = 4.85) receiving non-significantly different levels of noisy Sound from that of the deviant opponent (M = 5.4). Also in this case no significant relationship emerged with the covariate (Identity: F(1, 47) = 0.28, p = 0.60, η^2^_PARTIAL_ = 0.01).

### Ingroup vs Outgroup Deviant Reactions

**Fig 5:**
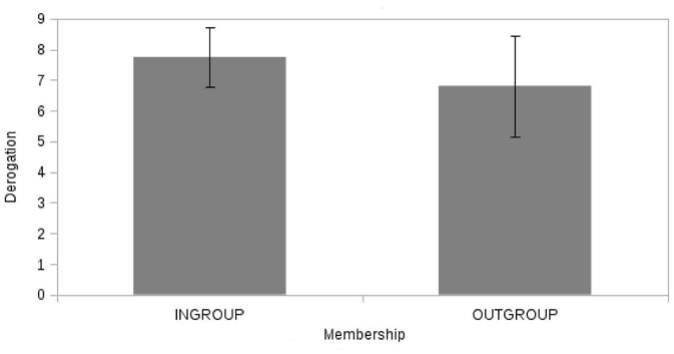
Derogation as function of membership (“Ingroup” vs. “Outgroup”).

**Fig 6:**
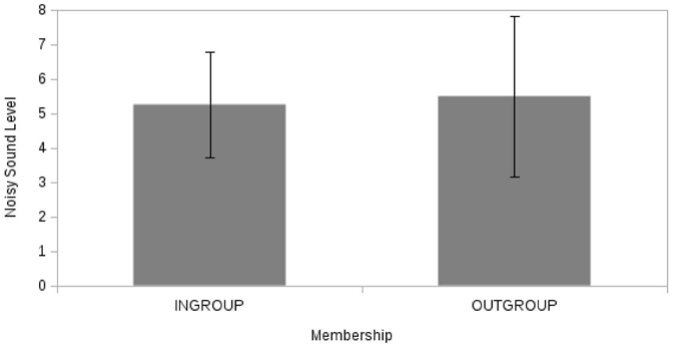
Noisy Sound Level as function of Membership (“Ingroup” vs. “Outgroup”).

With regards to the main effect of membership, we found a significant effect on the evaluation of the opponent (F(1, 46) = 6.0, p = 0.02, η^2^_PARTIAL_ = 0.12): the Ingroup deviant opponent (Fig 5) (H1) received a higher level of derogation (M = 7.7) than the Outgroup counterpart (M = 6.8). Also in this case none of the covariates had a significant effect (Identity: F(1, 46) = 0.13, p = 0.72, η^2^_PARTIAL_ = 0.003). On the contrary (H2b), the average aggressive behavior (level of noisy sound, Fig 6) did not differ as a function of membership of the deviant (H2b) (F(1, 46) = 0.16, p = 0.69, η^2^_PARTIAL_ = 0.003; M(Ingroup) = 5.25; M(Outgroup) = 5.50). No significant effects were reported for the covariate (Identity: F(1, 46) = 0.41, p = 0.53, η^2^_PARTIAL_ = 0.009).

### Blood Pressure

As control, the two membership groups (ingroup vs. outgroup) did not differ for both SBP and DBP or for MAP at the baseline (SBP t(47) = 1.2, p = 0.24; DBP t(47) = 0.45, p = 0.64; MAP t(47) = 0.74, p = .46). Since our main hypotheses concern the perceived threat from a deviant member, the following analysis will only consider the SBP levels. To verify the third hypothesis (H3), the effect of target manipulation was tested in ANOVA with the baseline SBP and gender as covariate. Due to problems with the device, 8 participants failed to take all the BP measurements. The effect was marginally significant (F (2, 39) = 2.92, p = 0.06, η^2^_PARTIAL_ = 0.136) with participants in the deviant condition reporting significantly (Sidak post-hoc test) higher SBP levels (M = 111.5) than the two normative conditions (respectively: M(normative1) = 109.2; M(normative2) = 108.2).

Finally (H4), to test if the relationship between SBP and the Reaction to the Deviant (defined as the noisy sound level given during the deviant condition of the target manipulation) (H4) observed in the Ingroup deviant condition differs from that observed in the Outgroup deviant condition, a multiple regression analysis was performed by regressing the SBP on the Reaction to the Deviant (defined as the noisy sound level given during the deviant condition of the target manipulation), on the Membership factor (ingroup vs. outgroup) and on their interaction. The baseline SBP, the gender and the deviant position were also introduced as covariates in the equation. All predictors were centered before entering the analysis and overall they explained about the 80% of the SBP variability (F(5,37) = 29.7, p<0.01). The Reaction to Deviant had a significant effect (b = -2.34, p = 0.02) as well as the interaction between the Reaction to Deviant and the Membership factor (b = 4.28, p = 0.01). While the direct effects of Membership alone (b = -1.59, p = 0.34), of gender (b = -0.97, p = 0.73), of the baseline SBP (b = 0.33, p = 0.16) and of deviant position (b = -1.09, p = 0.62) were not significant. Given that the interaction effect was significant, simple slope analysis was performed [43]. Results (Fig 5) clearly showed that for the Ingroup condition the relationship between the Reaction to the Deviant and the SBP was significant and negative (b = -4.38, p = 0.007) with participants respecting the norm (noisy sound levels below the limit of 8) reporting higher levels of SBP than participants breaking the norm. On the other hand, no significant relationship emerged in the Outgroup deviant condition (b = -0.10, p = 0.92).

## Discussion

The results of this study mainly support our hypotheses, and add new light to the literature on BSE on how to extend the manipulation of the deviant to a real group experimental context, and generalizes BSE to behavioral reactions to a deviant when a norm is salient. Results of the present study showed a BSE toward the ingroup anti-normative member, evaluating him more negatively with respect to the counterpart outgroup member. Furthermore, participants reported a medium-high level of identification with their group (M = 4.32, SD = 0.94), hence we could speculate that for all participants the unfair (ingroup/outgroup) behavior may represent a real threat to the group social identity.

With regards to the behavioral response to deviant members, as expected, results are in line with the interpretation that participants do not retaliate to the aggression toward both the ingroup or the outgroup counter-normative partners. Our explanation lies in the idea that the deviation of a member from a salient norm represents a threat to the positive image of the group, and that members are motivated to restore and reconsolidate this image [13]. For this reason they are impeded from using the “eye for eye, tooth for tooth” strategy for responding to a deviant. On the contrary, the mean comparisons showed that the aggressive reaction to the aggressive partner, both from ingroup or outgroup, is not dissimilar to that chosen when the partner plays normatively. This result allows us to speculate on the idea that participants did not endorse an aggressive (rule-breaking) reaction toward the deviants in their attempt to restore the norm by following the “do not be aggressive” rule. To substantiate this speculation, further investigations need to deeply analyze this idea using a different condition in which the priming of the norm by the researcher is not considered.

Finally, but nonetheless interesting, the results obtained on BP showed a significant increase in SBP when participants following the norm (i.e., to not use high levels of noisy sound) received unfair behavior from an ingroup member, but not when the same unfair behavior came from an outgroup deviant member. Based on past research, the lower levels of SBP in the outgroup deviant condition may be explained by the fact that, in this condition, participants are free from respecting a norm and may retaliate against the unfair behavior [36] [37] [38], whereas in the ingroup deviant condition participants are constrained by the norm to not retaliate even if they have the opportunity to do so; we think that this inhibition of the reaction may cause the higher levels of SBP. [27] Frings, Abrams, Randsley De Moura and Marques (2010) explained the arousal generated by an ingroup deviant in terms of the arousal: cost reward model (A:CR model) [44], where the higher the arousal, the higher the motivation of (and also the costs supported by) the other members of the group to react to the deviant in the attempt to reduce the arousal. These reactions include both actions that reduce (termed “inhibitors” such as the effort and energy involved or avoiding potential retaliation) or increase the tendency to act (termed “enablers,” such as maintaining the positive social identity or preventing other members from imitating the deviant action). The A:CR predictions may represent a valid explanation for the higher levels of SBP found in the ingroup deviant condition of our study, but also we cannot determine which of the actions (inhibitor or enabler) our participants adopted.

Another possible explanation may rely on findings, which concern the relation between cognitive effort and BSE [45]. In fact it has been shown that high-identified members invest more cognitive resources than less identified members in their attempts to exclude and to reclassify the deviant as atypical. Thus the higher levels of SBP we found could be related to the cognitive effort experienced by members when they try to exclude the ingroup deviant. Recent findings from Reese, Steffens, and Jonas (2013) [46] support this conclusion, suggesting that the extent of information processing mediates the BSE. These authors have shown that the information about deviant ingroup members require systematic processing while the outgroup deviant behavior is evaluated using heuristic processing. Consequentially, the higher levels of SBP we found in ingroup deviant may be explained in terms of cognitive effort enacted by ingroup members.

**Fig 7:**
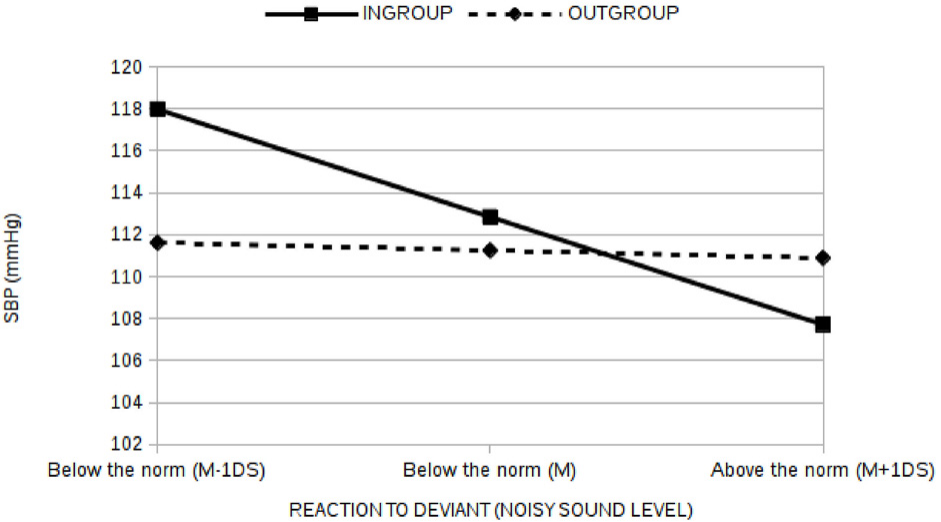
Moderation effects of Membership (Ingroup vs Outgroup) on the relationship between the Reaction to deviant (noisy Sound level) and the SBP (mmHg). The norm is to not use sound level higher than or equal to 8 (i.e. M+1SD, that corresponds to a Sound volume of about 100 Db). The average of noisy Sound level in the deviant condition was M = 6 (SD = 2).

We also observed that in the ingroup condition people differ in reacting to the unfair behavior: some people follow the norm notwithstanding the opponent’s unfair behavior and showing higher level of SBP than others who decided to break the rule and retaliate: in the outgroup condition such differences and effects on SBP did not emerge (Fig 7). Even though the relation between SBP and aggressive behavior is amply debated in literature [47], our results indicate that people successful in controlling their aggressive behavior in a minimal group setting (as a function of the momentarily salient norm and as of the membership of the deviant member), incur the possibly unhealthy consequence of increased state of arousal activation testified by a higher level of SBP. In this perspective, this result is coherent with that found in a another study [48] where it was found that basal cortisol is negatively correlated with the costly punishment of those individuals who did not contribute to a common group project (a typical social dilemma situation). The differential relationship between the (non-) aggressive behavior toward ingroup and outgroup deviants and the pressure levels may be explained in terms of greater conflicts involved in the decision to not punish ingroup deviants: the stronger the conflict between respecting the norm and retaliating against the unfair (rule-breaking) behavior, the lower the behavioral derogation and the higher the SBP [49].

### Main Strengths, Limitations and Future Research

Further studies focused on norm breaking are needed in order to determine in which condition the decision to derogate an ingroup member (who represents a threat for the group) is substituted by the decision to restore the norm at the cost of adopting a norm-breaking behavior (punish aggression by attacking). A recent study [50] suggests that people feel threatened if faced with the idea that they are non-prototypical and that this threat encourages especially high identifier members to use prototypicality as a standard for evaluating ingroup members. Hence, group members facing a deviant member have two choices: excluding him/her from the group because derogation is not enough to conform him/her to the group norms or, as an alternative like the one presented in our experimental paradigm, preferring to behave normatively following group norms (and not to further decrease the group identity) even if this means not punishing ingroup deviant members. In this latter case members would perhaps prefer using softer strategies, such as marginalizing deviant members, only when these methods do not damage the reputation of the group. In this light, maintaining the group’s values and norms is probably more important than the direct punishment of the deviant ingroup member.
